# Differences in phenotypes, symptoms, and survival in patients with cardiomyopathy—a prospective observational study from the Sahlgrenska CardioMyoPathy Centre

**DOI:** 10.3389/fcvm.2023.1160089

**Published:** 2023-04-17

**Authors:** C. Ljungman, E. Bollano, A. Rawshani, C. Nordberg Backelin, P. Dahlberg, I. Valeljung, M. Björkenstam, C. Hjalmarsson, M. Fu, T. Mellberg, S.-E. Bartfay, C. L. Polte, B. Andersson, N. Bergh

**Affiliations:** ^1^ Department of Molecular and Clinical Medicine, Institute of Medicine, University of Gothenburg, Gothenburg, Sweden; ^2^ Department of Cardiology, Sahlgrenska University Hospital, Gothenburg, Sweden; ^3^ Department of Clinical Physiology, Institute of Medicine, Sahlgrenska Academy, Gothenburg University, Gothenburg, Sweden; ^4^ Department of Transplantation, Sahlgrenska University Hospital, Gothenburg, Sweden

**Keywords:** cardiomyopathy, prognosis, survival, symptoms, heart failure

## Abstract

**Introduction:**

Cardiomyopathy is the fourth most common cause of heart failure. The spectrum of cardiomyopathies may be impacted by changes in environmental factors and the prognosis may be influenced by modern treatment. The aim of this study is to create a prospective clinical cohort, the Sahlgrenska CardioMyoPathy Centre (SCMPC) study, and compare patients with cardiomyopathies in terms of phenotype, symptoms, and survival.

**Methods:**

The SCMPC study was founded in 2018 by including patients with all types of suspected cardiomyopathies. This study included data on patient characteristics, background, family history, symptoms, diagnostic examinations, and treatment including heart transplantation and mechanical circulatory support (MCS). Patients were categorized by the type of cardiomyopathy on the basis of the diagnostic criteria laid down by the European Society of Cardiology (ESC) working group on myocardial and pericardial diseases. The primary outcomes were death, heart transplantation, or MCS, analyzed by Kaplan–Meier and Cox proportional regression, adjusted for age, gender, LVEF and QRS width on ECG in milliseconds.

**Results:**

In all, 461 patients and 73.1% men with a mean age of 53.6 ± 16 years were included in the study. The most common diagnosis was dilated cardiomyopathy (DCM), followed by cardiac sarcoidosis and myocarditis. Dyspnea was the most common initial symptom in patients with DCM and amyloidosis, while patients with arrhythmogenic right ventricular cardiomyopathy (ARVC) presented with ventricular arrythmias. Patients with ARVC, left-ventricular non-compaction cardiomyopathy (LVNC), hypertrophic cardiomyopathy (HCM), and DCM had the longest time from the debut of symptoms until inclusion in the study. Overall, 86% of the patients survived without heart transplantation or MCS after 2.5 years. The primary outcome differed among the cardiomyopathies, where the worst prognosis was reported for ARVC, LVNC, and cardiac amyloidosis. In a Cox regression analysis, it was found that ARVC and LVNC were independently associated with an increased risk of death, heart transplantation, or MCS compared with DCM. Further, female gender, a lower LVEF, and a wider QRS width were associated with an increased risk of the primary outcome.

**Conclusions:**

The SCMPC database offers a unique opportunity to explore the spectrum of cardiomyopathies over time. There is a large difference in characteristics and symptoms at debut and a remarkable difference in outcome, where the worst prognosis was reported for ARVC, LVNC, and cardiac amyloidosis.

## Introduction

Cardiomyopathies are a group of heterogenous diseases that affect the myocardium. The definition of a cardiomyopathy is a structurally and functionally abnormal heart muscle, in the absence of coronary artery disease, hypertension, valvular disease, and congenital heart disease sufficient to cause the observed myocardial abnormality (1). Cardiomyopathies are the fourth most common cause of heart failure globally after hypertension, ischemic heart disease, and chronic obstructive pulmonary disease (2). For unknown reasons, the overall prevalence of cardiomyopathies seems to increase (3). However, the true prevalence of cardiomyopathies among heart failure patients is doubtful (4). The possibility of diagnosing cardiomyopathies has increased because of an increasing access to non-invasive diagnostic tools.

The spectrum of symptoms, clinical characteristics, and survival in patients with cardiomyopathies ranges from subclinical signs without symptoms to acute heart failure requiring intensive care, mechanical support, and in some patients, heart transplantation (5). The prognosis of cardiomyopathies may be influenced by modern pharmacological and device treatment of heart failure with reduced ejection fraction (HFrEF), heart transplantation, and anti-inflammatory treatment but to what extent is uncertain (4, 6, 7). Changes in environmental factors such as those caused by COVID-19 (8) and the prevalence of obesity are among factors that may influence the spectrum of cardiomyopathies (9). Previous work with cardiomyopathy registries did not include all subtypes of cardiomyopathies but rather focused on certain forms, mainly hypertrophic cardiomyopathy (HCM) (10, 11). Thus, detailed studies of symptoms, clinical presentation, patient characteristics, diagnostic examinations, and prognosis among all different types of cardiomyopathies are largely lacking.

Therefore, a prospective comprehensive study, called the Sahlgrenska CardioMyoPathy Centre (SCMPC) study, including all cardiomyopathies, also rare subtypes, was initiated. The aim was to study the present spectrum of cardiomyopathies, clinical symptoms, diagnostic examinations and survival, as well as future changes in cardiomyopathies by building a prospective clinical observational cohort that includes all patients referred to a tertiary center for a suspected cardiomyopathy.

## Methods

### Study population

The SCMPC study is a prospective observational study that was initiated in 2018 and is ongoing. It is being conducted at the Sahlgrenska University Hospital in Gothenburg, Sweden. The Sahlgrenska University Hospital is a tertiary center with a main catchment area of 2.0 million inhabitants in the county Västra Götalandsregionen (VGR). Patients can also be referred from hospitals outside of VGR predominantly from the surrounding regions of Halland, Småland, Dalsland, and Värmland, in all covering the main western part of Sweden. The Sahlgrenska University Hospital is one of the two centers in Sweden that performs heart transplantation.

Patients were enrolled prospectively following informed consent. The inclusion criteria were patients ≥18 years, with a suspected cardiomyopathy irrespective of subtype. The patients were included in three different ways: *de novo* patients referred for an evaluation of suspected cardiomyopathy after 1 January 2018, patients with chronic disease visiting the outpatient clinic at the tertiary center because of a diagnosis of cardiomyopathy, and heart-transplanted cardiomyopathy patients followed up at our center.

Information from medical records before 2018 was also collected if the date of evaluation and diagnosis had been made before 2018. The current study included all patients, along with follow-up on outcomes, in the SCMPC database until 15 August 2022.

### Baseline and follow-up data

At inclusion, the following baseline data were collected: date of referral and evaluation, symptoms and date of debut, medical background, coexisting conditions, family history regarding known first-grade relatives with confirmed cardiomyopathy, laboratory data, blood pressure, pharmacological treatment at inclusion, biomarkers, and whether any genetic testing had been performed during the course of clinical practice. Information regarding alcohol and drug abuse were collected from medical records and laboratory blood samples, and this included information about the presence of Phosphatidylethanol in blood (B-Peth) if available and deemed clinically significant by the senior consultant cardiologist concerned. However, B-Peth was not mandatory and not registered in the database. Information on radiation in the thoracic area and treatment with cytotoxic drugs prior to the debut of symptoms was collected from the medical records of all patients, as well as signs of significant infections in association with the debut of cardiomyopathy, which were deemed clinically significant by the senior consultant cardiologist concerned.

Data on invasive and non-invasive examinations such as right heart catheterization (RHC), endomyocardial biopsy, cardiopulmonary exercise test (CPET), imaging with echocardiography, cardiac magnetic resonance (CMR), 18F-fluorodeoxyglucose positron emission tomography/computed tomography (FDG-PET/CT), myocardial perfusion scintigraphy, 3,3-diphosphono-1,2-propanodicarboxylic acid (DPD) scintigraphy, coronary angiography, and computed tomography (CT) were collected from the medical records. In the present analysis, relevant examinations performed within 182 days after onset of symptoms are presented. The date of diagnosis and type of cardiomyopathy were registered. Diagnosis on the type of cardiomyopathy was made by the senior consultant cardiologist concerned on the basis of current ESC recommendations. If uncertainty on diagnosis existed, the relevant case was discussed by the members of the heart failure team to reach a consensus. Ischemic heart disease was excluded along with coronary angiography or CT if appropriate, and patients with heart failure caused by ischemic heart disease or hypertension were not included.

Data on treatment, biomarkers, imaging and interventions (such as heart transplantation and MCS), and death were updated in the medical records during follow-up on a yearly basis. The SCMPC database was built on a Microsoft Access database platform. A complete list of variables in the database is included in Supplementary Table S1.

### Cardiomyopathies

The European Society of Cardiology (ESC) international classification system divides cardiomyopathies into five main groups on the basis of the patient's clinical state and imaging findings. These groups are as follows: arrhythmogenic right ventricular cardiomyopathy (ARVC), dilated cardiomyopathy (DCM), HCM, restrictive cardiomyopathy, and unclassified cardiomyopathy, consisting of left-ventricular non-compaction cardiomyopathy (LVNC) and Takotsubo cardiomyopathy. Inflammatory cardiomyopathies are a subgroup of DCM, divided into three major groups: infectious, immune-mediated, and toxic myocarditis (1). In the present database, inflammatory cardiomyopathies were grouped under myocarditis (viral, bacterial, immune-mediated, or toxic), cardiac sarcoidosis (CS), and giant cell myocarditis (GCM). Peripartum cardiomyopathy is a subgroup of DCM in the ESC international classification system, but in the present database, it is recorded separately. Cardiac amyloidosis is a form of restrictive cardiomyopathy, but in this database, cardiac amyloidosis was grouped separately, primarily because of the new possibility for specific treatment. Further, the type of amyloidosis was subgrouped in systemic AL amyloidosis, wild-type transthyretin amyloidosis (TTRw), hereditary transthyretin amyloidosis, and other forms of amyloidosis but analyzed together.

Upon diagnosis, all patients were grouped in the following categories: ARVC, cardiac amyloidosis, CS, DCM, GCM, HCM, LVNC, and myocarditis. Patients diagnosed with Takotsubo cardiomyopathy, restrictive cardiomyopathy, peripartum cardiomyopathy, and unspecified cardiomyopathy not fulfilling the diagnostic criteria for certain diagnoses were placed in the category of “other” in the present study. If a diagnosis of cardiomyopathy was ruled out after evaluation, the patient came under the category of “non-confirmed cardiomyopathy”.

### Blood samples and biobank

Upon entry in the study, venous blood samples were collected and stored in a regional biobank. These samples were collected either in the peripheral vein or in the central vein during RHC if appropriate. Three blood samples were collected in three different tubes: EDTA anticoagulated whole blood for DNA extraction, EDTA anticoagulated plasma sample, and serum. The serum and plasma samples were centrifugated in 2,000*g* for 10 min and aliquoted with robot. All samples were stored in −80°C. If there was a clinical indication to perform endomyocardial biopsy, 1–2 biopsies were collected and saved in a biobank. The biopsies were snap-frozen in liquid nitrogen and thereafter stored in −80°C. Endomyocardial biopsies were obtained before heart transplantation.

All samples were handled by the Regional Biobank.

### Statistical methods

The study participants' baseline characteristics are presented as mean and median, along with standard deviation (SD) or frequencies. No hypotheses tests were used to compare baseline characteristics since there were no explicit hypotheses related to these. Instead, standardized mean differences to compare the distributions among baseline variables were used. Survival distribution among different cardiomyopathies was studied using the Kaplan–Meier method, and group differences were compared by using the log-rank test. Patients who underwent heart transplantation before inclusion were excluded in the survival analysis. The primary outcome was a composite endpoint consisting of death, heart transplantation, or mechanical circulatory support (MCS). The Cox proportional hazards model was used to study the association between the type of cardiomyopathy and the composite outcome adjusted for clinical predictors [age, sex, ejection fraction (EF) at debut and QRS width in milliseconds on ECG]. Age, EF at debut, and QRS width in milliseconds were analyzed as continuous variables. DCM was used as the reference group. Data on vital status were obtained from the Population Registry, and medical records and data on heart transplantation were obtained from the patients’ medical record. Outcome was calculated as the time from inclusion to the first event of MCS, heart transplantation, or death. Schoenfeld residuals were used to assess the assumption of proportional hazards, which was fulfilled in each model. Missing data were imputed using a chained random forest. Briefly, each variable was imputed using a random forest with all other predictors as covariables. The algorithm iterates multiple times over all variables until no further improvement in the average out-of-bag prediction error is achieved (Michael Meyer https://cran.r-project.org/web/packages/missRanger/vignettes/missRanger.html) All statistical analyses were performed by using RStudio version 4.2.2.

## Ethical considerations

The SCMPC study obtained ethical approval from the Swedish Ethical Review Authority (Dnr: 935–17; 2018). Upon inclusion, all patients were de-identified and given an individual study code.

## Results

### Patient characteristics

In all, 461 patients, a majority being men, with a mean age of 53.6 ± 16 years were included. The most common cardiomyopathy was DCM, followed by CS and myocarditis (Table 1). In 41 patients, the diagnosis of cardiomyopathy could not be confirmed. Of the patients with amyloidosis, 12 (44.4%) were diagnosed with systemic AL amyloidosis, 12 (44.4%) with TTRw amyloidosis, 2 (7.4%) with hereditary amyloidosis, and 1 (3.7%) with another type of amyloidosis, which was not specified. Patients with myocarditis were generally younger (35 ± 15years), whereas patients with cardiac amyloidosis were older (71 ± 7 years) and had substantially more comorbidities. Hypertension was the most common co-existing condition present in 27.6% of all patients. A first-grade relative with a confirmed diagnosis of cardiomyopathy was most prevalent in patients with LVNC (28.5%), HCM (28.3%), and ARVC (27.0%), followed by patients with DCM (4.3%). Accordingly, genetic testing was most frequently performed in ARVC (54.5%), HCM (43.4%), and DCM (6.2%). Endomyocardial biopsy was most frequently performed in patients with GCM (66.7%), CS (65.2%), and amyloidosis (59.3%). A high consumption of alcohol was numerically more common in patients with DCM (Table 1).

**Table 1:** Baseline characteristics of 461 patients with cardiomyopathy.

	Overall	DCM	Sarcoidosis	Myocarditis	HCM	Non-confirmed	Amyloidosis	ARVC	LVNC	GCM	Other
Patients	461	161	69	56	53	41	27	11	7	6	30
Age in years mean ± SD	53.6 (16.0)	54.6 (14.6)	58.2 (9.0)	34.6 (15.2)	54.7 (15.1)	55.4 (14.1)	70.6 (7.5)	52.4 (20.3)	55.8 (16.1)	64.4 (8.1)	50.6 (15.0)
Female *n* (%)	124 (26.9)	41 (25.5)	16 (23.2)	13 (23.2)	22 (41.5)	12 (29.3)	4 (14.8)	4 (36.4)	3 (42.9)	2 (33.3)	7 (23.3)
SBP (mmHg) mean ± SD	128.4 (23.3)	125.5 (22.1)	134.5 (23.3)	120.1 (14.9)	135.9 (20.7)	137.8 (24.9)	143.6 (38.7)	105.0 (49.5)	NA (NA)	114.4 (20.3)	128.5 (20.5)
Smoking *n* (%)	31 (10.5)	15 (13.2)	3 (7.1)	3 (9.1)	5 (17.2)	2 (7.1)	1 (6.7)	0 (0.0)	0 (0.0)	1 (20.0)	1 (5.9)
LVEF (%) mean ± SD	42.7 (17.3)	25.1 (11.3)	50.0 (11.1)	54.9 (7.1)	60.9 (6.1)	52.1 (13.9)	56.8 (11.1)	52.0 (5.7)	Na (NA)	33.0 (18.2)	46.0 (14.7)
Endomyocardial biopsy performed *n* (%)	167 (36.2)	55 (34.2)	45 (65.2)	14 (25.0)	7 (13.2)	13 (31.7)	16 (59.3)	1 (9.0)	0 (NA)	4 (66.7)	12 (40.0)
**Comorbidities**											
Hypertension *n* (%)	116 (27.6)	36 (24.7)	24 (41.4)	5 (9.4)	14 (29.2)	14 (35.9)	13 (52.0)	0 (0.0)	1 (16.7)	2 (40.0)	7 (23.3)
Diabetes mellitus *n* (%)	43 (10.1)	15 (10.1)	10 (16.4)	1 (1.9)	4 (8.3)	5 (12.8)	4 (16.7)	0 (0.0)	1 (16.7)	0 (0.0)	3 (10.0)
COPD *n* (%)	6 (1.4)	3 (2.1)	0 0.0)	2 (3.8)	1 (2.1)	0 (0.0)	(0.0)	0 (0.0)	0 (0.0)	0 (0.0)	0 (0.0)
Stroke or TIA *n* (%)	15 (4.2)	8 (6.6)	2 (3.8)	0 (0.0)	0 (0.0)	1 (3.0)	3 (20.0)	0 (0.0)	0 (0.0)	0 (0.0)	1 (4.2)
IHD *n* (%)	19 (4.6)	6 (4.1)	2 (3.3)	1 (1.9)	4 (9.3)	2 (5.1)	2 (8.7)	0 (0.0)	0 (0.0)	0 (0.0)	2 (6.7)
Rheumatic disease *n* (%)	24 (5.8)	5 (3.4)	8 (13.6)	1 (1.9)	2 (4.2)	4 (10.5)	2 (8.7)	0 (0.0)	0 (0.0)	1 (20.0)	1 (3.4)
Chronic kidney disease *n* (%)	20 (4.7)	4 (2.7)	2 (3.2)	1 (1.9)	4 (8.3)	1 (2.6)	5 (20.8)	0 (0.0)	0 (0.0)	0 (0.0)	3 (10.0)
ACHD *n* (%)	4 (1.0)	0 (0.0)	0 (0.0)	1 (1.9)	1 (2.2)	0 (0.0)	0 (0.0)	0 (0.0)	1 (16.7)	0 (0.0)	1 (3.3)
Syndrome *n* (%)	2 (0.5)	1 (0.7)	0 (0.0)	0 (0.0)	1 (2.1)	0 (0.0)	0 (0.0)	0 (0.0)	0 (0.0)	0 (0.0)	0 (0.0)
Bechers disease *n* (%)	1 (0.3)	1 (0.9)	0 (0.0)	0 (0.0)	0 (0.0)	0 (0.0)	0 (0.0)	0 (0.0)	0 (0.0)	0 (0.0)	0 (0.0)
Myositis *n* (%)	2 (0.6)	0 (0.0)	0 (0.0)	1 (2.6)	0 (0.0)	1 (3.4)	0 (0.0)	0 (0.0)	0 (0.0)	0 (0.0)	0 (0.0)
**Toxic effects before onset of symptoms**											
Alcohol *n* (%)	49 (14.1)	27 (20.3)	5 (10.0)	5 (12.5)	5 (13.5)	3 (11.5)	0 (0.0)	0 (0.0)	0 (0.0)	1 (25.0)	3 (12.5)
Narcotics *n* (%)	11 (2.9)	8 (5.7)	0 (0.0)	1 (2.2)	2 (4.8)	0 (0.0)	0 (0.0)	0 (0.0)	0 (0.0)	0 (0.0)	0 (0.0)
Anabolic steroid *n* (%)	2 (0.5)	1 (0.7)	0 (0.0)	0 (0.0)	1 (2.4)	0 (0.0)	0 (0.0)	0 (0.0)	0 (0.0)	0 (0.0)	0 (0.0)
Malignancy *n* (%)	25 (6.1)	14 (9.7)	1 (1.7)	0 (0.0)	1 (2.3)	1 (2.8)	4 (16.0)	1 (9.1)	0 (0.0)	0 (0.0)	3 (10.0)
Treatment with cytotoxic drugs *n* (%)	14 (3.4)	6 (4.2)	1 (1.7)	0 (0.0)	1 (2.3)	0 (0.0)	2 (7.7)	1 (10.0)	0 (0.0)	0 (0.0)	3 (10.0)
Radiation toward thoracic area *n* (%)	3 (0.7)	3 (2.1)	0 (0.0)	0 (0.0)	0 (0.0)	0 (0.0)	0 (0.0)	0 (0.0)	0 (0.0)	0 (0.0)	0 (0.0)
Infection at onset *n* (%)	45 (11.1)	17 (11.7)	4 (6.8)	19 (39.6)	1 (2.3)	2 (5.6)	1 (4.2)	0 (0.0)	0 (0.0)	0 (0.0)	1 (3.4)
**Heritance**											
First-grade relative with confirmed cardiomyopathy *n* (%)	29 (6.3)	7 (4.3)	0 (0.0)	1 (1.6)	15 (28.3)	0 (0.0)	0 (0.0)	3 (27.0)	2 (28.5)	0 (NA)	1 (3.3)
Genetic testing performed *n* (%)	41 (8.9)	10 (6.2)	0 (0.0)	0 (0.0)	23 (43.4)	0 (0.0)	1 (3.7)	6 (54.5)	0 (NA)	0 (NA)	1 (3.3)

Values presented as means ± standard deviation or frequencies (%) as appropriate. ACHD, adult congenital heart disease; ARVC, arrhythmogenic right-ventricular cardiomyopathy; COPD, chronic obstructive pulmonary disease; DCM, dilated cardiomyopathy; GCM, giant cell myocarditis; HCM, hypertrophic cardiomyopathy; IHD, ischemic heart disease; NA, not available; LVEF, left ventricular ejection fraction; LVNC, left-ventricular non-compaction cardiomyopathy. Other including diagnoses Takotsubo, Restrictive, Peripartum cardiomyopathy, and unspecified cardiomyopathy not fully fulfilling diagnostic criteria for certain diagnoses. SBP, systolic blood pressure; TIA, transient ischemic attack.

### Symptoms

Symptoms at debut varied depending to some extent on the type of cardiomyopathy. Dyspnea was the most common symptom at onset in the cohort (63.3%), followed by fatigue (51.9%) and chest pain (30.5%). Dyspnea was present at debut in 85% of patients with DCM and in 81.8% of patients with amyloidosis. Chest pain was the most common symptom at debut in patients with myocarditis (82.7%) and GCM (80%). In patients with ARVC, ventricular arrythmias was the most common symptom at debut and present in 70% of the patients. Patients with GCM (60%) experienced ventricular arrythmias at debut (Table 2).

**Table 2:** Symptoms at debut in 461 patients with cardiomyopathy.

	Overall	DCM	Sarcoidosis	Myocarditis	HCM	Non-confirmed	Amyloidosis	ARVC	LVNC	GCM	Other
	461	161	69	56	53	41	27	11	7	6	30
Dyspnea *n* (%)	235 (63.3)	110 (85.3)	33 (60.0)	15 (31.2)	21 (53.8)	13 (40.6)	18 (81.8)	2 (33.3)	5 (71.4)	3 (60.0)	15 (53.6)
Chest pain *n* (%)	108 (30.5)	19 (16.2)	8 (15.7)	43 (82.7)	9 (27.3)	11 (35.5)	5 (22.7)	1 (14.3)	2 (28.6)	4 (80.0)	6 (20.7)
Fatigue *n* (%)	176 (51.9)	85 (75.2)	27 (50.9)	18 (38.3)	11 (34.4)	5 (16.1)	10 (52.6)	1 (14.3)	5 (83.3)	3 (75.0)	11 (40.7)
Edema *n* (%)	51 (15.1)	33 (30.6)	2 (3.6)	1 (2.1)	2 (6.1)	1 (3.3)	7 (31.8)	0 (0.0)	2 (40.0)	0 (0.0)	3 (11.1)
Syncope *n* (%)	50 (13.7)	10 (7.9)	11 (19.3)	3 (6.1)	10 (27.0)	8 (25.8)	1 (4.5)	2 (25.0)	0 (0.0)	0 (0.0)	5 (18.5)
Atrial fibrillation *n* (%)	43 (11.4)	16 (12.5)	6 (10.5)	1 (2.0)	3 (8.1)	4 (12.1)	7 (28.0)	2 (28.6)	1 (14.3)	2 (40.0)	1 (3.6)
Ventricular premature contractions *n* (%)	34 (9.3)	15 (11.9)	6 (10.7)	2 (4.2)	1 (2.7)	6 (19.4)	0 (0.0)	1 (14.3)	0 (0.0)	0 (0.0)	3 (11.5)
Ventricular Tachycardia *n* (%)	51 (13.8)	14 (11.2)	13 (22.4)	1 (2.0)	2 (5.4)	5 (16.7)	1 (4.3)	7 (70.0)	0 (0.0)	3 (60.0)	5 (17.9)
Bradycardia *n* (%)	39 (10.8)	4 (3.3)	18 (32.7)	4 (8.0)	1 (2.7)	7 (22.6)	2 (8.7)	0 (0.0)	0 (0.0)	1 (25.0)	2 (7.1)
Unspecified arrythmia *n* (%)	73 (19.9)	21 (17.1)	13 (22.8)	6 (12.5)	6 (16.2)	13 (40.6)	3 (13.0)	5 (62.5)	1 (20.0)	0 (0.0)	5 (17.2)
Cardiac arrest *n* (%)	16 (4.3)	8 (6.2)	3 (5.2)	1 (2.0)	1 (2.9)	0 (0.0)	0 (0.0)	1 (12.5)	0 (0.0)	0 (0.0)	2 (6.9)

Values presented as frequencies (%). ARVC, arrhythmogenic right-ventricular cardiomyopathy; DCM, dilated cardiomyopathy; GCM, giant cell myocarditis; HCM, hypertrophic cardiomyopathy; LVNC, left-ventricular non-compaction cardiomyopathy; Other including diagnoses Takotsubo, Restrictive, Peripartum cardiomyopathy, and unspecified cardiomyopathy not fully fulfilling diagnostic criteria for certain diagnoses.

### Time from debut of symptoms until inclusion

**Figure 1:**
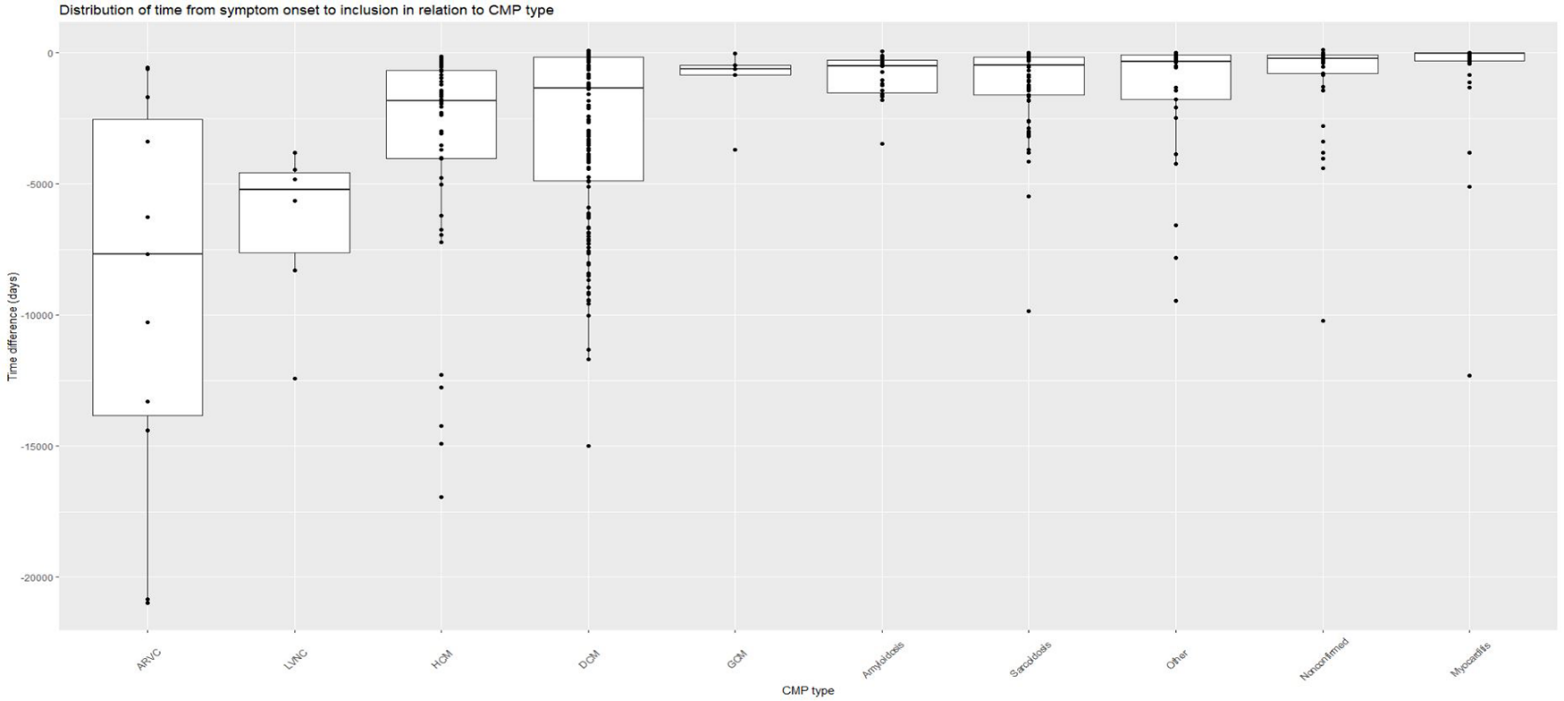
Time from debut of symptoms until inclusion in SCMPC by CMP data in days difference between the date of debut of symptoms until the date of inclusion in the SCMPC database presented with a Box and whiskers plot with median, upper, and lower quartiles and minimum and maximum values by CMP. Each patient presented as a data point with a dot. Sorted by CMP in descending order by days. ARVC, arrhythmogenic right-ventricular cardiomyopathy; CMP, cardiomyopathy; DCM, dilated cardiomyopathy; GCM, giant cell myocarditis; HCM, hypertrophic cardiomyopathy; LVNC, left-ventricular non-compaction cardiomyopathy. Other including diagnoses Takotsubo, Restrictive, Peripartum cardiomyopathy, and unspecified cardiomyopathy not fully fulfilling diagnostic criteria for certain diagnoses.

There was a difference in time from debut of symptoms until inclusion in the SCMPC study where patients with LVNC, ARVC, HCM, and DCM had the longest time from debut of symptoms until inclusion. Patients with myocarditis and where cardiomyopathy could not be confirmed had the shortest time from debut of symptoms until inclusion. With regard to myocarditis, most patients were included in the acute phase; however, five (9%) were included in the chronic postmyocarditis phase >2,500 days since debut of symptoms (Figure 1).

### Outcome

In all, 14 (3%) patients died, and 81 (17.6%) underwent heart transplantation during follow-up. No patient was lost to follow-up. A majority of those who died was found to have suffered from cardiac amyloidosis (*n* = 9). Most patients who underwent heart transplantation had a diagnosis of DCM; however, patients with ARVC and LVNC had the highest number of heart transplants (Table 3).

**Table 3:** Outcome in 461 patients with cardiomyopathy.

Outcome	Overall	DCM	Sarcoidosis	Myocarditis	HCM	Non-confirmed	Amyloidosis	ARVC	LVNC	GCM	Other
*n* (%)	461	161	69	56	53	41	27	11	7	6	30
Death (%)	14 (3.0)	1 (0.6)	1 (1.4)	0 (0.0)	1 (1.9)	1 (2.4)	9 (33.3)	0 (0.0)	0 (0.0)	0 (0.0)	1 (3.3)
Heart transplantation (%)	81 (17.6)	48 (29.8)	7 (10.1)	1 (1.8)	7 (13.2)	0 (0.0)	2 (7.4)	7 (63.6)	4 (57.1)	1 (16.7)	4 (13.3)
MCS (%)	15 (3.3)	10 (6.2)	1 (1.4)	0 (0.0)	0 (0.0)	0 (0.0)	0 (0.0)	0 (0.0)	1 (14.3)	1 (16.7)	2 (6.7)

Values presented as frequencies (%). ARVC, arrhythmogenic right-ventricular cardiomyopathy; DCM, dilated cardiomyopathy; GCM, giant cell myocarditis; HCM, hypertrophic cardiomyopathy; LVNC, left-ventricular non-compaction cardiomyopathy; MCS, mechanical circulatory support; Other including diagnoses Takotsubo, Restrictive, Peripartum cardiomyopathy, and unspecified cardiomyopathy not fully fulfilling diagnostic criteria for certain diagnoses.

**Figure 2:**
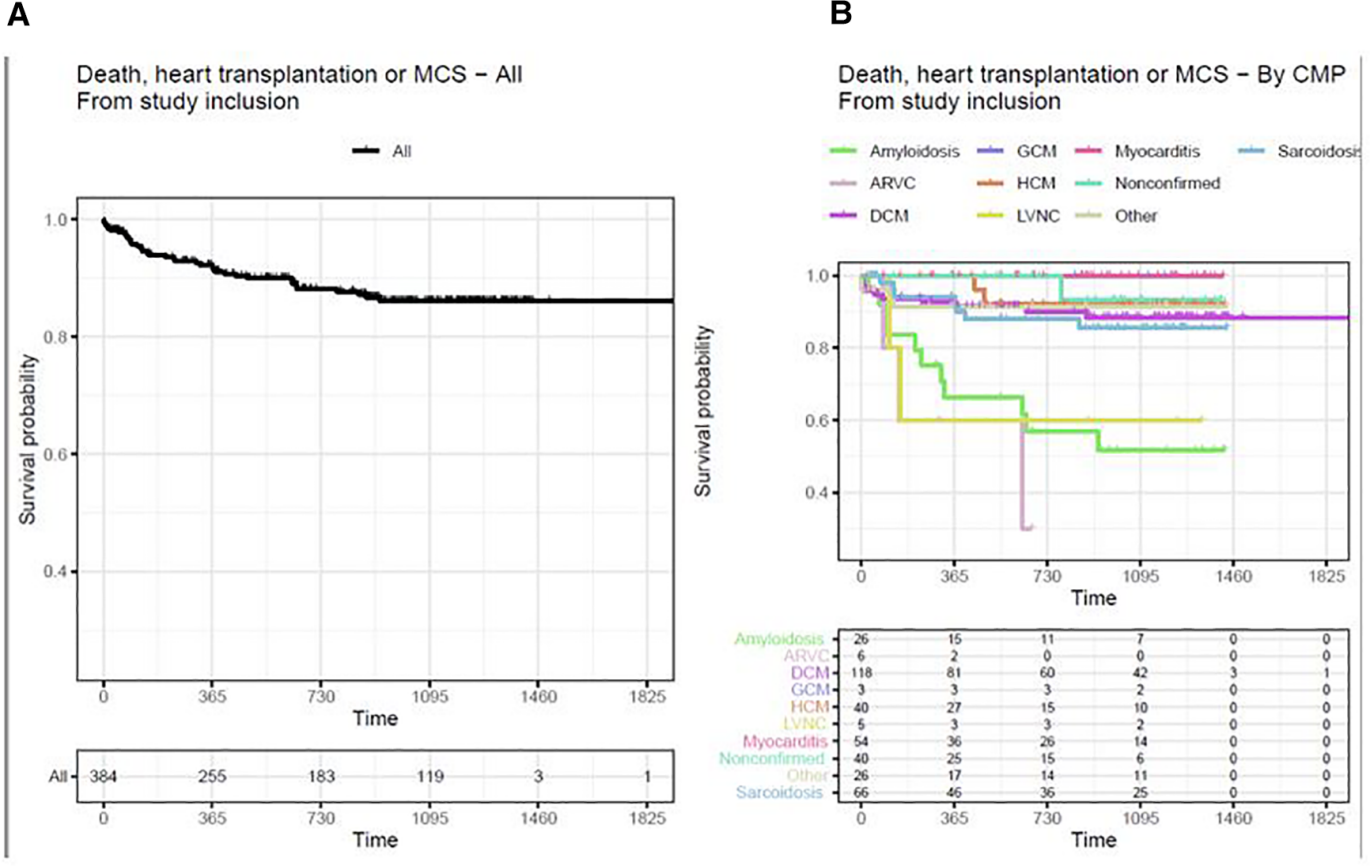
(**A**) Death, heart transplantation, or MCS in all cardiomyopathies from study inclusion. (**B**) Death, heart transplantation, or MCS from study inclusion by CMP-type Kaplan–Meier curves with outcome variables of death, heart transplantation, or MCS. Time is measured in days. Patients transplanted before study inclusion are excluded from the analysis. ARVC, arrhythmogenic right-ventricular cardiomyopathy; CMP, cardiomyopathy; DCM, dilated cardiomyopathy; GCM, giant cell myocarditis; HCM, hypertrophic cardiomyopathy; LVNC, left-ventricular non-compaction cardiomyopathy. Other including diagnoses Takotsubo, Restrictive, Peripartum cardiomyopathy, and unspecified cardiomyopathy not fully fulfilling diagnostic criteria for certain diagnoses.

It was found that after 2.5 years from the start of the study, 86.2% of the patients had survived and had not been subject to heart transplantation or MCS (Figure 2A). There was a difference in outcome depending on the type of cardiomyopathy, where the worst prognosis was reported in patients with amyloidosis, ARVC, and LVNC (Figure 2B). The highest survival rates were reported for myocarditis and non-confirmed cardiomyopathy.

### Cox regression model of death, heart transplantation, or MCS

**Figure 3:**
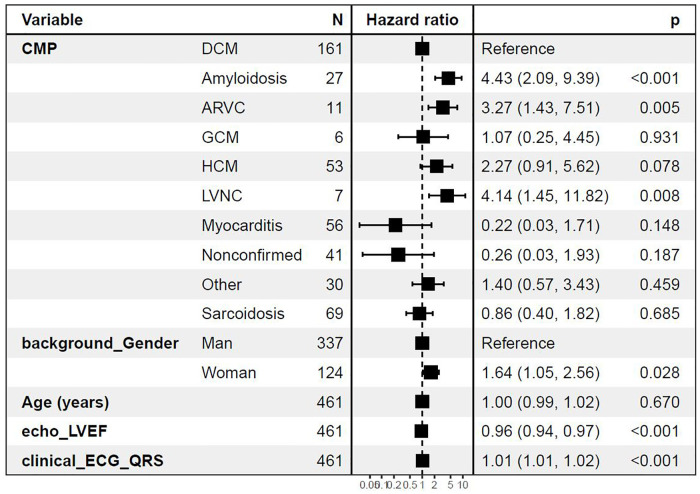
Cox regression analysis with diagnoses as predictors for death, heart transplantation, or MCS Cox regression adjusted for age, gender, left ventricular ejection fraction (LVEF), and QRS width on ECG in milliseconds at baseline. ARVC, arrhythmogenic right-ventricular cardiomyopathy; CMP, cardiomyopathy; DCM, dilated cardiomyopathy; GCM, giant cell myocarditis; HCM, hypertrophic cardiomyopathy; LVNC, left-ventricular non-compaction cardiomyopathy. Other including diagnoses Takotsubo, Restrictive, Peripartum cardiomyopathy, and unspecified cardiomyopathy not fully fulfilling diagnostic criteria for certain diagnoses.

Risk of death, heart transplantation, or MCS was compared among different cardiomyopathies and adjusted for differences in age, gender, and ejection fraction with DCM as the reference group. Myocarditis showed the lowest risk, while amyloidosis, LVNC, and ARVC showed the highest risk of the primary outcome compared with DCM. Excluding the cases of chronic myocarditis (*n* = 5), defined as cases with >2,500 days since debut of symptoms, resulted in the hazard ratio approaching zero because of the lack of events in the myocarditis group. Hence, the myocarditis group experienced a very low risk of the outcomes studied in the Cox model. Further, female gender, lower EF, and wider QRS width on ECG were independently associated with an increased risk of the primary endpoint (Figure 3).

## Discussion

The establishment of the comprehensive clinical SCMPC study allowed us to perform studies regarding patient phenotype, symptoms, and survival. We found notable differences in patient characteristics, symptoms, and outcomes related to the subtypes of cardiomyopathies.

### Outcome

Our results show a difference in outcome where ARVC, LVNC, and cardiac amyloidosis carry an increased risk of death, heart transplantation, or MCS, while patients with myocarditis and non-confirmed cardiomyopathy carry the lowest risk. When adjusted for differences in age, gender, and ejection fraction, amyloidosis, LVNC, and ARVC were independently associated with the primary outcome compared with patients with DCM. Further, female gender, lower ejection fraction, and wider QRS duration on ECG were associated with a worse prognosis.

ARVC is an inherited heterogenous disease initially believed to affect only the right ventricle, with fibrofatty infiltration, but is now supposed to frequently involve both ventricles (12). Presentation with heart failure at debut of symptoms is rare, and accordingly in the SCMPC database, the symptom at debut was ventricular tachycardia in 70% of patients (12). Data from the Nordic ARVC registry show that 17% of patients underwent heart transplantation from 1988 until 2014, and the indication for heart transplantation was heart failure in 90% of them (13). Although the SCMPC database included only a limited number of patients with ARVC, the severity of the diagnosis compared with other cardiomyopathies is evident.

Left ventricular non-compaction is characterized by a bilayered myocardium with prominent trabeculations and was classified as a primary cardiomyopathy in 2006 by the American Heart Association (14), while the ESC working group describes LVNC as an unspecified cardiomyopathy (1). In the SCMPC study, 28.5% of patients with LVNC have a confirmed first-grade relative with cardiomyopathy, which corresponds well to the fact that LVNC is an inherited disease in 20%–40% of patients (15). The prognosis is heterogenous, but in symptomatic patients with LVNC, the 6-year mortality rate is 75% from presentation (15). It has been suggested that LVNC poses a higher risk compared with DCM, which can be confirmed in the SCMPC database (16). However, due to the fact that a small number of patients with LVNC are included in this database, caution should be exercised in the interpretation of the results.

Our results show a worse prognosis in patients with cardiac amyloidosis, which is expected, because it is a progressive infiltrative cardiomyopathy mainly affecting the elderly (17), and for which there has been a lack of specific treatment all along. Patients with cardiac amyloidosis have, to a lesser extent, been considered suitable candidates for heart transplantation mainly because of age and comorbidities, which possibly explains the findings in the SCMPC study. Specific disease-modifying treatments that can delay or halt amyloid deposition have recently been introduced, and the creation of the SMPC database provides the basis for evaluating the effects of specific disease-modifying treatments with respect to cardiac function and survival (18).

In the present analysis, the female gender was associated with an increased risk of death, heart transplantation, or MCS. This finding is puzzling to some extent, because most studies have shown a lower mortality rate in women with DCM, for example, and in women referred for an evaluation of advanced heart failure (19, 20). This finding will be investigated in future studies. A lower EF and a wider QRS width on ECG were both independently associated with worse prognosis. These measurements are frequently available and could be used in the assessment of the clinical risk in patients with cardiomyopathy.

### The panorama of cardiomyopathies

The panorama of cardiomyopathies included in the SCMPC database is on expected lines, with DCM being the most prevalent cardiomyopathy (5). However, the second most common cardiomyopathy found in the SCMPC database is CS. Cardiac sarcoidosis and GCM are rare but severe forms of inflammatory cardiomyopathies, which might be two expressions of the same disease, with the exception that GCM has a more fulminant clinical course (21). Sarcoidosis is more common in the Nordic countries, which may explain the high frequency rate of CS (22). However, it is estimated that only 5% of patients with sarcoidosis develop symptomatic CS. Another reason for the relatively high number of CS patients might be an increased awareness and the use of investigational measures. Several ongoing projects on CS within the SCMPC study will hopefully increase our knowledge about the disease.

Hypertrophic cardiomyopathy is the most common inherited cardiomyopathy, but in the SCMPC database, it is the fourth most common type of cardiomyopathy. This may be explained by the fact that only those who are refractory to treatment and subject to an evaluation of intervention, for example alcohol septal ablation or heart transplantation, are referred to a tertiary center. Similarly, patients with peripartum cardiomyopathy are few in number, since the maternity clinic is located at only one of the hospitals within the Sahlgrenska University Hospital (Östra) and only the most severely ill patients in need of referral to a tertiary clinic is included in the SCMPC database. Likewise, only a small number of patients with Takotsubo cardiomyopathy, also known as stress cardiomyopathy, are included (23). Most patients with Takotsubo cardiomyopathy report restoration of cardiac function and are not admitted for evaluation, and hence, are not included in the SCMPC study.

### Symptoms and time to inclusion

Our results show a diversity in patients’ characteristics and symptoms. The diversity in characteristics is expected as cardiomyopathies are a heterogenous group of diseases. Our results show that dyspnea—a fundamental symptom of heart failure—is the most common symptom at debut for DCM and cardiac amyloidosis. However, no patient with ARVC presented with dyspnea at debut, but rather most frequently presented with ventricular tachycardia. The differences in symptoms at debut may lead to a better understanding of differential diagnosis early in the evaluation of patients with suspected cardiomyopathy (24). Further, there is a large difference in the time from debut of symptoms until inclusion in the SCMPC study, where inherited diseases such as ARVC, LVNC, and HCM have a longer time from debut of symptoms until inclusion compared with myocarditis.

### Limitations

There are some important limitations to address in this study. The SCMPC study builds on patients referred to a tertiary center, which lends itself to a selection bias when compared with the entire population of patients with cardiomyopathies. Patients referred to a tertiary center include those severely ill and in need of evaluation for heart transplantation and MCS, which results in a cohort not representative of all patients with cardiomyopathies. The nature of this observational study also exposes it to the risk of survival bias. Further, some cardiomyopathy patients are included in small numbers, which will result in a low inclusion rate. Patients are included upon signing an informed consent form, thus indicating that patients not willing to participate in the SCPMC study will not be included, although the numbers are very few. There are missing values on variables that need to be addressed. An assessment of alcohol and drug consumption was made by the senior consultant cardiologist, and no specific amounts were found in the patients.

In conclusion, the development of the SCMPC study including all types of cardiomyopathy patients referred to the tertiary center provides the opportunity to investigate cardiomyopathies over a period of time. Our results show a large difference in characteristics and symptoms at debut. Further, our results show a remarkable difference in outcomes, where the worst prognosis was found for ARVC, LVNC and cardiac amyloidosis. However, the overall survival rate obtained from study inclusion was found to be high, with 86% of patients having survived without heart transplantation or MCS after 2.5 years. This SCMPC study offers a unique opportunity to explore the full spectrum of cardiomyopathies over a period of time.

## Data Availability

The raw data supporting the conclusions of this article will be made available by the authors without undue reservation.
