# Supplementary material for: Differences in phenotypes, symptoms, and survival in patients with cardiomyopathy—a prospective observational study from the Sahlgrenska CardioMyoPathy Centre
Source: Front Cardiovasc Med. 2023 Apr 17;10:1160089. doi: 10.3389/fcvm.2023.1160089 (PMC10150027; doi:10.3389/fcvm.2023.1160089)
Supplement: Supplementary file 1 [file Datasheet1.pdf]

Supplementary Material

Differences in phenotypes, symptoms, and survival in patients with cardiomyopathy – a prospective observational study from the Sahlgrenska Cardiomyopathy Centre (SCMPC)

Ljungman C[1,2*], Bollano E[1,2], Rawshani A[1,2], Nordberg Backelin C[1,2], Dahlberg P[1,2], Valeljung I[1,2], Björkenstam M[1,2], Hjalmarsson C[1,2], Fu M[1], Mellberg T[1,2], Bartfay S-E[1,2], Polte CL[3, 4], Andersson B[1,2], Bergh N[1, 2, 5]

*** Correspondence:** Charlotta Ljungman: charlotta.ljungman@vgregion.se

List of all variables used in the SCMPC study

Background variables

background_AFibHistory

background_Afib

background_Aflut

background_Age

background_Alkohol

background_AnabSter

background_AoValvue

background_Biobank_blod

background_Biobank_tissue

background_Bradycardia

background_CardArrest

background_Chestpain

background_CMPDiagn

background_Comment

background_Cytotoxicity

background_CytotxSpecific

background_Diabetes

background_Dyspnea

background_Edema

background_EmbStroke

background_FirstContact

background_Gender

background_GUCH

background_GUCH_Spec

background_Hypertension

background_Infection

background_Info_cons

background_InfSpec

background_IschHD

background_COPD

background_Malignancy

background_MaignancylSpecific

background_MiValvue

background_Musculardisease

background_Narcotics

background_No_symptom

background_OthAbuse

background_OtherArrhymiaHist

background_OthEndocrine

background_OthEndocrineSpec

background_OthMuscSpec

background_OthSymptoms

background_OthToxocity

background_OthTxSpec

background_PhysiscianContact

background_RadiationThorax

background_Referral

background_RegistrationDate

background_RegistrationUnit

background_Renaldisease

background_Rheumaticdisease

background_Smokeing

background_SpecCMPT

background_SpecRheum

background_StrokeTIA

background_SympDebut

background_Syncope

background_Syndrome

background_SyndrSpeific

background_Fatigue

background_TriValvue

background_UnspecArrh

background_VPC

background_VT

background_VTHistory

Examinations

cath_Biobank_Tissue

cath_CathDat

cath_CO

cath_Hist_Spec

cath_Histologi

cath_Left_Biopsy

cath_Left_Cath

cath_NO_inhal

cath_Pam

cath_PCWP

cath_Ram

cath_Right_Biopsy

cath_Right_Cath

cath_VirusPCR

cath_Work

echo_CVT

echo_Echo_find

echo_EchoDat

echo_IVSD

echo_LA_Area

echo_LA_Vol

echo_LV_Diast_Dysfunction

echo_LVEDD

echo_LVEDV

echo_LVEF

echo_LVESD

echo_LVESV

echo_LVM

echo_MI

echo_PWD

echo_RV_Dilat

echo_RV_Dysfunction

echo_RV_SP

echo_TI

eecg_AECG

eecg_AECGArr

eecg_AECGBPDr

eecg_AECGDat

eecg_AECGIsch

eecg_AECGVO2

eecg_AECGW

mri_LGE

mri_LGE_Spec

mri_LVEDV

mri_LVEF

mri_LVESV

mri_LVM

mri_MRI_find

mri_MRIDat

mri_RVEDV

mri_RVEF

mri_RVESV

petct_PETCT

petct_PETCTDat

petct_PETCTKomm

petct_PETCTRes

Clinical data

clinical_Alive

clinical_ClinicDat

clinical_DBP

clinical_ECG_AVB

clinical_ECG_HR

clinical_ECG_LBBB

clinical_ECG_QRS

clinical_ECG_Rhythm

clinical_HF_Ascites

clinical_HF_Edema

clinical_HF_Jugular

clinical_HF_Rales

clinical_HF_Shock

clinical_Length

clinical_MortSpec

clinical_NYHA

clinical_SBP

clinical_Weight

Family

family_1aGradSibl

family_1aGradSibl_

family_1aGS_Number

family_1aGS_Relation

family_1aGS_PrevDis_Number

family_1aGS_PrevDis_Relat

family_Fam_Dis

family_Gentest

family_GenVar_Spec

family_Cascad

family_NoRelative

family_OthSibl

family_OthSibl_Antal

family_OthSibl_Relat

family_Syndr_Spec

family_Syndrom

family_UppdatDat

family_UtfallGentest

Laboratory

laboratory_AutoAK

laboratory_Biobank_blood

laboratory_BNP

laboratory_CK

laboratory_CRP_max

laboratory_Hb

laboratory_Crea

laboratory_LabComm

laboratory_LabDat

laboratory_NT-proBNP

laboratory_TNI_max

laboratory_TNT_max

Medications

medications_AAR

medications_ACEARB

medications_ACO

medications_ARNI

medications_BBL

medications_IMS

medications_Inotropi

medications_Loop

medications_MedDat

medications_MRA

medications_SGLT2i

Outcome

date_death

date_death_tpl

date_death_tpl_mcs

date_mcs

date_tpl

diff_symp_inklusion

event_death

event_death_tpl

event_death_tpl

event_mcs

event_tpl

surv_death

surv_death_symp

surv_death_tpl

surv_death_tpl_mcs

S1 Supplement table 1. Heart failure medications in all patients with cardiomyopathy

Medication	Overall	DCM	Sarcoidosis	Myocarditis	HCM	Nonconfirmed	Amyloidosis	ARVC	LVNC	GCM	Other
n	461	161	69	56	53	41	27	11	7	6	30
ACEi or ARBs n(%)	162 (35.1)	71 (44.0)	32 (46.4)	13 (23.2)	9 (17.0)	13 (31.7)	9 (33.3)	1 (9.1)	0 (NA)	4 (66.7)	10 (33.3)
Beta blocker n(%)	227 (49.2)	104 (64.6)	37 (53.6)	8 (14.3)	30 (56.6)	11 (26.8)	11 (40.7)	6 (90.9)	0 (NA)	4 (66.7)	16 (53.3)
Mineral receptor antagonist n(%)	113 (24.5)	67 (41.6)	17 (24.6)	1 (1.8)	4 (7.5)	7 (17.0)	11 (40.7)	1 (9.1)	0 (NA)	1 (16.7)	4 (13.3)
Angiotensin receptor Neprilysin inhibitor n(%)	35 (7.6)	29 (18.0)	4 (5.8)	0 (NA)	0 (NA)	1 (2.4)	0 (NA)	0 (NA)	0 (NA)	0 (NA)	1 (3.3)
Sodium Glucose cotransporter-2 inhibitor n(%)	23 (5.0)	17 (10.6)	4 (5.8)	0 (NA)	1 (1.9)	1 (2.4)	0 (NA)	0 (NA)	0 (NA)	0 (NA)	0 (NA)
Loop diuretics n(%)	107 (23.2)	63 (39.1)	13 (18.8)	1 (1.8)	5 (9.4)	3 (7.3)	14 (51.9)	0 (NA)	0 (NA)	3 (50.0)	5 (16.7)

Values presented as frequencies (%). ACE Angiotensin converting enzyme inhibitor, ARB Angiotensin receptor blockers, ARVC Arrhythmogenic Right-Ventricular Cardiomyopathy, DCM Dilated Cardiomyopathy, GCM Giant Cell Myocarditis, HCM Hypertrophic Cardiomyopathy, LVNC Left-ventricular Non-Compaction Cardiomyopathy, Other including diagnoses Takotsubo, Restrictive, Peripartum cardiomyopathy and unspecified cardiomyopathy not fully fulfilling diagnostic criteria for certain diagnosis.

Supplement table 2

Table 2. Baseline characteristics of 456 patients with cardiomyopathy, excluding patients with chronic myocarditis

	Overall	DCM	Sarcoidosis	Myocarditis	HCM	Nonconfirmed	Amyloidosis	ARVC	LVNC	GCM	Other
n	456	161	69	51	53	41	27	11	7	6	30
Age in years mean±SD	53.7 (15.9)	54.6 (14.6)	58.2 (9.0)	33.6 (14.8)	54.7 (15.1)	55.4 (14.1)	70.6 (7.5)	52.4 (20.3)	55.8 (16.1)	64.4 (8.1)	50.6 (15.0)
Female n(%)	123 (27.0)	41 (25.5)	16 (23.2)	12 (23.5)	22 (41.5)	12 (29.3)	4 (14.8)	4 (36.4)	3 (42.9)	2 (33.3)	7 (23.3)
SBP (mmHg) mean±SD	128.4 (23.3)	125.5 (22.1)	134.5 (23.3)	120.1 (14.9)	135.9 (20.7)	137.8 (24.9)	143.6 (38.7)	105.0 (49.5)	NA (NA)	114.4 (20.3)	128.5 (20.5)
Smoking n(%)	31 (10.6)	15 (13.2)	3 (7.1)	3 (10.0)	5 (17.2)	2 (7.1)	1 (6.7)	0 (0.0)	0 (0.0)	1 (20.0)	1 (5.9)
LVEF (%) mean±SD	43.4 (17.5)	25.9 (11.8)	49.0 (11.6)	54.6 (7.0)	61.9 (10.3)	52.9 (12.4)	54.5 (11.4)	51.3 (8.2)	52.5 (10.6)	37.4 (18.6)	46.3 (13.2)

Table 2. Baseline characteristics of 456 patients with cardiomyopathy, excluding patients with chronic myocarditis

	Overall	DCM	Sarcoidosis	Myocarditis	HCM	Nonconfirmed	Amyloidosis	ARVC	LVNC	GCM	Other
Endomyocardial biopsy n(%)	166 (87.8)	55 (80.9)	45 (95.7)	13 (92.9)	7 (87.5)	13 (81.2)	16 (100.0)	1 (100.0)	0 (NaN)	4 (80.0)	12 (85.7)
**Comorbidites**											
Hypertension n(%)	116 (27.8)	36 (24.7)	24 (41.4)	5 (10.2)	14 (29.2)	14 (35.9)	13 (52.0)	0 (0.0)	1 (16.7)	2 (40.0)	7 (23.3)
Diabetes mellitus n(%)	43 (10.2)	15 (10.1)	10 (16.4)	1 (2.0)	4 (8.3)	5 (12.8)	4 (16.7)	0 (0.0)	1 (16.7)	0 (0.0)	3 (10.0)
COPD n(%)	6 (1.4)	3 (2.1)	0 (0.0)	2 (4.1)	1 (2.1)	0 (0.0)	0 (0.0)	0 (0.0)	0 (0.0)	0 (0.0)	0 (0.0)
Stroke or TIA n(%)	15 (4.2)	8 (6.6)	2 (3.8)	0 (0.0)	0 (0.0)	1 (3.0)	3 (20.0)	0 (0.0)	0 (0.0)	0 (0.0)	1 (4.2)

Table 2. Baseline characteristics of 456 patients with cardiomyopathy, excluding patients with chronic myocarditis

	Overall	DCM	Sarcoidosis	Myocarditis	HCM	Nonconfirmed	Amyloidosis	ARVC	LVNC	GCM	Other
IHD n(%)	19 (4.6)	6 (4.1)	2 (3.3)	1 (2.1)	4 (9.3)	2 (5.1)	2 (8.7)	0 (0.0)	0 (0.0)	0 (0.0)	2 (6.7)
Rheumatic disease n(%)	24 (5.8)	5 (3.4)	8 (13.6)	1 (2.1)	2 (4.2)	4 (10.5)	2 (8.7)	0 (0.0)	0 (0.0)	1 (20.0)	1 (3.4)
Chronic kidney disease n(%)	20 (4.8)	4 (2.7)	2 (3.2)	1 (2.0)	4 (8.3)	1 (2.6)	5 (20.8)	0 (0.0)	0 (0.0)	0 (0.0)	3 (10.0)
ACHD n(%)	4 (1.0)	0 (0.0)	0 (0.0)	1 (2.0)	1 (2.2)	0 (0.0)	0 (0.0)	0 (0.0)	1 (16.7)	0 (0.0)	1 (3.3)
Syndrome n(%)	2 (0.5)	1 (0.7)	0 (0.0)	0 (0.0)	1 (2.1)	0 (0.0)	0 (0.0)	0 (0.0)	0 (0.0)	0 (0.0)	0 (0.0)
**Muscular disease**											
Bechers disease n(%)	1 (0.3)	1 (0.9)	0 (0.0)	0 (0.0)	0 (0.0)	0 (0.0)	0 (0.0)	0 (0.0)	0 (0.0)	0 (0.0)	0 (0.0)

Table 2. Baseline characteristics of 456 patients with cardiomyopathy, excluding patients with chronic myocarditis

	Overall	DCM	Sarcoidosis	Myocarditis	HCM	Nonconfirmed	Amyloidosis	ARVC	LVNC	GCM	Other
Myositis n(%)	2 (0.7)	0 (0.0)	0 (0.0)	1 (2.9)	0 (0.0)	1 (3.4)	0 (0.0)	0 (0.0)	0 (0.0)	0 (0.0)	0 (0.0)
**Toxic effects before onset of symptoms**											
Alcohol n(%)	49 (14.2)	27 (20.3)	5 (10.0)	5 (13.5)	5 (13.5)	3 (11.5)	0 (0.0)	0 (0.0)	0 (0.0)	1 (25.0)	3 (12.5)
Narcotics n(%)	11 (2.9)	8 (5.7)	0 (0.0)	1 (2.3)	2 (4.8)	0 (0.0)	0 (0.0)	0 (0.0)	0 (0.0)	0 (0.0)	0 (0.0)
Anabolic steroids n(%)	2 (0.5)	1 (0.7)	0 (0.0)	0 (0.0)	1 (2.4)	0 (0.0)	0 (0.0)	0 (0.0)	0 (0.0)	0 (0.0)	0 (0.0)
Malignancy n(%)	25 (6.1)	14 (9.7)	1 (1.7)	0 (0.0)	1 (2.3)	1 (2.8)	4 (16.0)	1 (9.1)	0 (0.0)	0 (0.0)	3 (10.0)
Cytotoxicity n(%)	14 (3.5)	6 (4.2)	1 (1.7)	0 (0.0)	1 (2.3)	0 (0.0)	2 (7.7)	1 (10.0)	0 (0.0)	0 (0.0)	3 (10.0)

Table 2. Baseline characteristics of 456 patients with cardiomyopathy, excluding patients with chronic myocarditis

	Overall	DCM	Sarcoidosis	Myocarditis	HCM	Nonconfirmed	Amyloidosis	ARVC	LVNC	GCM	Other
Radiation toward thoracic area n(%)	3 (0.8)	3 (2.1)	0 (0.0)	0 (0.0)	0 (0.0)	0 (0.0)	0 (0.0)	0 (0.0)	0 (0.0)	0 (0.0)	0 (0.0)
Radiation toward thoracic area(%)	44 (11.0)	17 (11.7)	4 (6.8)	18 (40.9)	1 (2.3)	2 (5.6)	1 (4.2)	0 (0.0)	0 (0.0)	0 (0.0)	1 (3.4)
**Heritance**											
First-grade relative with confirmed cardiomyopathy n(%)	29 (31.2)	7 (17.5)	0 (0.0)	1 (20.0)	15 (78.9)	0 (0.0)	0 (0.0)	3 (75.0)	2 (66.7)	0 (NaN)	1 (14.3)
Genetic testing performed n(%)	41 (52.6)	10 (34.5)	0 (0.0)	0 (0.0)	23 (95.8)	0 (0.0)	1 (33.3)	6 (100.0)	0 (NaN)	0 (NaN)	1 (14.3)
**Heart failure medications**											

Table 2. Baseline characteristics of 456 patients with cardiomyopathy, excluding patients with chronic myocarditis

	Overall	DCM	Sarcoidosis	Myocarditis	HCM	Nonconfirmed	Amyloidosis	ARVC	LVNC	GCM	Other
ACE or ARB n(%)	161 (51.1)	71 (67.0)	32 (58.2)	12 (28.6)	9 (26.5)	13 (54.2)	9 (42.9)	1 (16.7)	0 (NaN)	4 (80.0)	10 (45.5)
Beta blocker n(%)	227 (70.9)	104 (93.7)	37 (67.3)	8 (19.0)	30 (88.2)	11 (45.8)	11 (52.4)	6 (100.0)	0 (NaN)	4 (80.0)	16 (72.7)
Mineral receptor antagonist n(%)	113 (35.4)	67 (61.5)	17 (30.9)	1 (2.4)	4 (11.8)	7 (29.2)	11 (50.0)	1 (16.7)	0 (NaN)	1 (20.0)	4 (18.2)
Angiotensin receptor neprilysin inhibitor n(%)	35 (11.1)	29 (26.9)	4 (7.3)	0 (0.0)	0 (0.0)	1 (4.2)	0 (0.0)	0 (0.0)	0 (NaN)	0 (0.0)	1 (4.5)
Sodium Glucose cotransporter-2 inhibitor n(%)	23 (25.8)	17 (65.4)	4 (33.3)	0 (0.0)	1 (6.7)	1 (9.1)	0 (0.0)	0 (0.0)	0 (NaN)	0 (0.0)	0 (0.0)
Loop diuretics n(%)	106 (33.2)	63 (57.3)	13 (23.6)	0 (0.0)	5 (15.2)	3 (12.5)	14 (63.6)	0 (0.0)	0 (NaN)	3 (60.0)	5 (22.7)

Values presented as means ± standard deviation or frequencies (%) as appropriate. ACE Angiotensin converting enzyme inhibitor, ARB Angiotensin receptor blockers, ACHD Adult congenital heart disease, ARVC Arrhythmogenic Right-Ventricular Cardiomyopathy, COPD Chronic obstructive pulmonary disease, DCM Dilated Cardiomyopathy, GCM Giant Cell Myocarditis, HCM Hypertrophic Cardiomyopathy, IHD ischemic heart disease, NA not available, LVEF Left ventricular ejection fraction, LVNC Left-ventricular Non-Compaction Cardiomyopathy, Other including diagnoses Takotsubo, Restrictive, Peripartum cardiomyopathy and unspecified cardiomyopathy not fully fulfilling diagnostic criteria for certain diagnosis, SBP systolic blood pressure, TIA transient ischemic attack.
